# The Current Diagnostic Accuracy on Free Peritoneal Fluid in Computed Tomography to Determinate the Necessity of Surgery in Blunt Bowel and Mesenteric Trauma—Systemic Review and Meta-Analysis

**DOI:** 10.3390/diagnostics11112028

**Published:** 2021-11-02

**Authors:** Szu-An Chen, Chen-Yu Wang, Chih-Po Hsu, Jia-Yen Lin, Chi-Tung Cheng, Chun-Hsiang Ouyang, Jen-Fu Huang, Chien-Hung Liao

**Affiliations:** 1 Department of Traumatology and Emergency Surgery, Chang Gung Memorial Hospital, Chang Gung University, Taoyuan 33328, Taiwan; wacamama@cgmh.org.tw (S.-A.C.); m7831@cgmh.org.tw (C.-P.H.); meditator184@gmail.com (J.-Y.L.); atong89130@gmail.com (C.-T.C.); detv090@gmail.com (C.-H.O.); m7626@cgmh.org.tw (J.-F.H.); 2 Department of Surgery, Linkou Chang Gung Memorial Hospital, Chang Gung University, Taoyuan 33328, Taiwan; wanglayla8151@gmail.com

**Keywords:** traumatic bowel and mesenteric injury, computed tomography, free peritoneal fluid, systematic review

## Abstract

Traumatic bowel mesenteric injury (TBMI) is a challenge in trauma care. The presence of free peritoneal fluid (FF) in computed tomography (CT) was considered the indication for surgical intervention. However, conservative treatment should be applied for minor injuries. We conduct a systematic review to analyze how reliable the FF is to assess the TBMI. Publications were retrieved by structured searching among databases, review articles and major textbooks. For statistical analysis, summary receiver operating characteristic curves (SROCs) were computed using hierarchical models. Fourteen studies enrolling 4336 patients were eligible for final qualitative analysis. The SROC line was created by a hierarchical summary receiver operating characteristic model. The summary sensitivity of FF to predict surgical TBMI was 0.793 (95% CI: 0.635–0.894), and the summary specificity of FF to predict surgical TBMI was 0.733 (95% CI: 0.468–0.896). The diagnostic odds ratio was 10.531 (95% CI: 5.556–19.961). This study represents the most robust evidence (level 3a) to date that FF is not the absolute but an acceptable indicator for surgically important TBMI. However, there is still a need for randomized controlled trials to confirm.

## 1. Introduction

Traumatic mesenteric injury (TBMI) is a challenging setting in trauma care. After blunt trauma, bowel and mesenteric injuries are infrequent, reported among 5% of abdominal trauma, but they are nevertheless dangerous. TBMI causes significant blood loss from disrupted mesenteric vessels immediately and remotely from injury time. Furthermore, the disruption of blood flow will lead to bowel ischemia, necrosis, and the discontinuity of the wall with eventual delayed rupture or ischemic strictures [1,2,3]. The physical presentation and examinations of these injuries may be subtle and are often overshadowed by other injuries, resulting in a clinical diagnostic dilemma. Unrecognized bowel and mesenteric injuries may account for high morbidity and mortality [4,5,6,7]. Therefore, surgical intervention, at least with laparoscopic exploration, became the leading strand when the TBMI was suspicious. On the other hand, non-therapeutic surgical intervention has also been associated with increased patient morbidity and increased hospital stay [4,8,9,10,11,12].

The contrast-enhanced computed tomography (CT) has an essential diagnostic modality for TBMI [4,8,9,10,11,12]. The CT presentation also localizes anatomic sites of TBMI and guides angiographic or surgical intervention. Previous studies presented free peritoneal fluid indicating the surgically important TBMI [12,13,14,15]. However, CT’s various performance in diagnosing surgically important TBMI was reported to be between 11–98% [13,14,16,17,18,19,20,21]. Overdiagnosis of the surgical TBMI resulted in numerous non-therapeutic surgeries. Therefore, a fine selection of candidates for surgical intervention became critical.

In this study, we reviewed the literature in English to provide a thorough evaluation of the current state of the art of FF in CT in diagnosing surgically important TBMI. The authors also performed a meta-analysis and diagnostic accuracy summary to determine the significance of FF in CT.

## 2. Materials and Methods

### 2.1. Literature Search

The systematic literature search was performed based on the preferred reporting items for systematic reviews and meta-analyses (PRISMA) [22]. The study was registered in Prospero CRD42021256035. The MEDLINE, EMBASE, Web of Knowledge, and Google Scholar databases were searched using the following keywords: traumatic bowel injury, intestinal injury, bowel perforation, mesenteric injury, mesentery trauma, computed tomography, free fluid, hemoperitoneum, organ, lesion, laceration, rupture, trauma, parenchyma, injury, abdominal trauma. The bibliographies of relevant articles were also interrogated to identify further studies. Inclusion criteria included original studies investigating study samples with TBMI under CT diagnosis. Traumatic mesenteric injuries are defined as an injury of the mesentery, mesocolon, and supplying vessels of hollow abdominal viscus, where the organ is directly injured from trauma and needs resection, repair, or control of bleeding for definitive treatment, or where hollow viscus injury due to direct trauma is detected by CT and managed conservatively. Only English language articles, or articles where there was an online English translation available, were included. Because of the advance of technology, the diagnostic performance of CT improved during the past decades. We only searched the literature from 2010 to 2019. Authors whose names appeared on multiple studies that were otherwise eligible for inclusion were contacted to avoid any data duplication.

In a second retrieval phase, references in the original papers were examined for other publications according to the above terms, and these related articles were accessed. This procedure was repeated in a further two phases. An extensive manual search was also performed among publications and textbooks on the diagnosis and treatment of TBMI. Image review and registry reviews were excluded. The studies with non-adult or non-human subjects were excluded from this review.

### 2.2. Data Collection and Validity Assessment

After limiting the initial search based on the inclusion and exclusion criteria, studies were shortlisted based on the title and abstract information. Two independent reviewers (Chen-Yu. Wang and Szu-An Chen) screened the abstracts, and full-text publications were reviewed if insufficient information in the abstract was deemed suitable for inclusion. Extracted data were entered into a pre-prepared database independently by both reviewers and compared at the end for consistency. Data extraction was performed by using spreadsheet software (Excel; Microsoft, Redmond, Wash). Any differences in opinion regarding inclusions were discussed with a third party (Chi-Tung Cheng). Data were extracted from the studies using a data extraction sheet. The Quality Assessment for Diagnostic Accuracy

Studies 2nd version (QUADAS2) tool consisted of 14 items and a checklist was used for quality assessment by two reviewers (as shown in supplementary materials).

### 2.3. Statistical Analysis

Statistics included the sensitivity and specificity of computed tomography in TBI. A diagnostic test’s accuracy is derived from the sum of all true-positive and true-negative findings divided by the study size. Summary sensitivity and specificity, with 95% confidence intervals (95% CI), and summary receiver operating characteristic (SROC) plots were estimated using the hierarchical summary receiver operating characteristic models. The statistical analyses were performed with open-source Review Manager, version 5.3 (the Nordic Cochrane Centre, the Cochrane Collaboration, Copenhagen, Denmark, 2014). The parameter of SROC was performed with MetaDTA: Diagnostic Test Accuracy Meta-Analysis version 1.45 [23].

## 3. Results

### 3.1. Search Strategy

**Figure 1:**
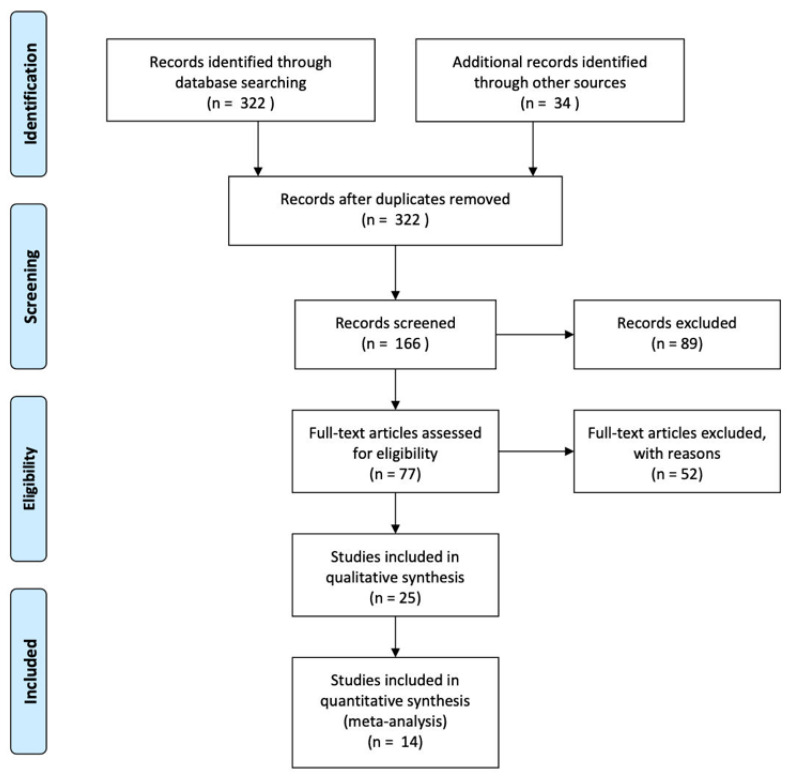
The flow diagram of this review.

The systematic review yielded a total of 322 studies using the search terms. We excluded the publications before 2010 because the technology and resolution of CT was disparate from the current status of these diagnostic tools. A total of 166 studies were included for full-text analysis. After reviewing the abstract, 89 articles were excluded due to no non-trauma related injury, pediatric group study, no English-based works of literature, reviews, or no full-text available as Figure 1.

### 3.2. Study Characteristics

Twenty-six articles that present the FF in CT to diagnose TBMI were included. There was one prospective study and twenty-five retrospective studies. Free peritoneal fluid was the typical presentation in CT of TBMI with a high occurrence rate and an acceptable diagnostic characteristic. The fourteen manuscripts listed in Table 1 contained appropriate data for analysis. The meta-analysis was performed for the summary ROC curve (SROC). As shown in Table 2, of 4336 patients scanned by computed tomography, 290 patients had the true positive diagnosis of surgically important TBMI. However, the high false positive diagnosis was made in 516 patients, and false negative occurred in 157 patients. The surgically important TBMI were 447 and accounted for 10.7%.

**Figure 2:**
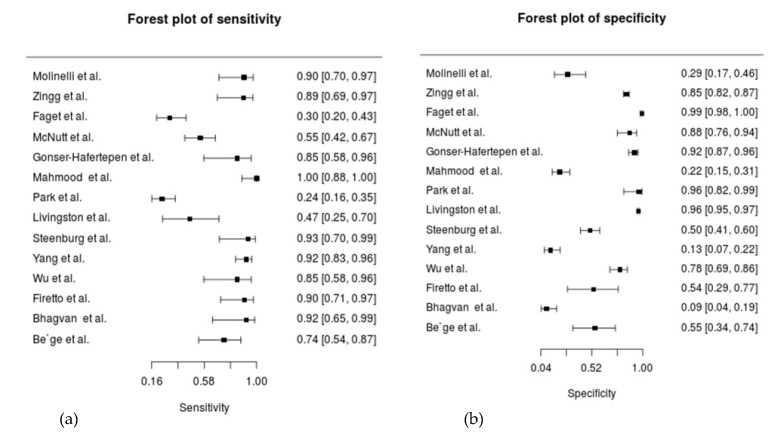
The forest plot of included studies; (**a**) the forest plot of sensitivity of 14 studies; (**b**) the forest plot of sensitivity of 14 studies.

The sensitivity of CT in diagnosing TBMI ranged from 24–100%, and the specificity ranged from 9–99%. The forest plot of included studies was presented in Figure 2.

### 3.3. Meta-Analysis

**Figure 3:**
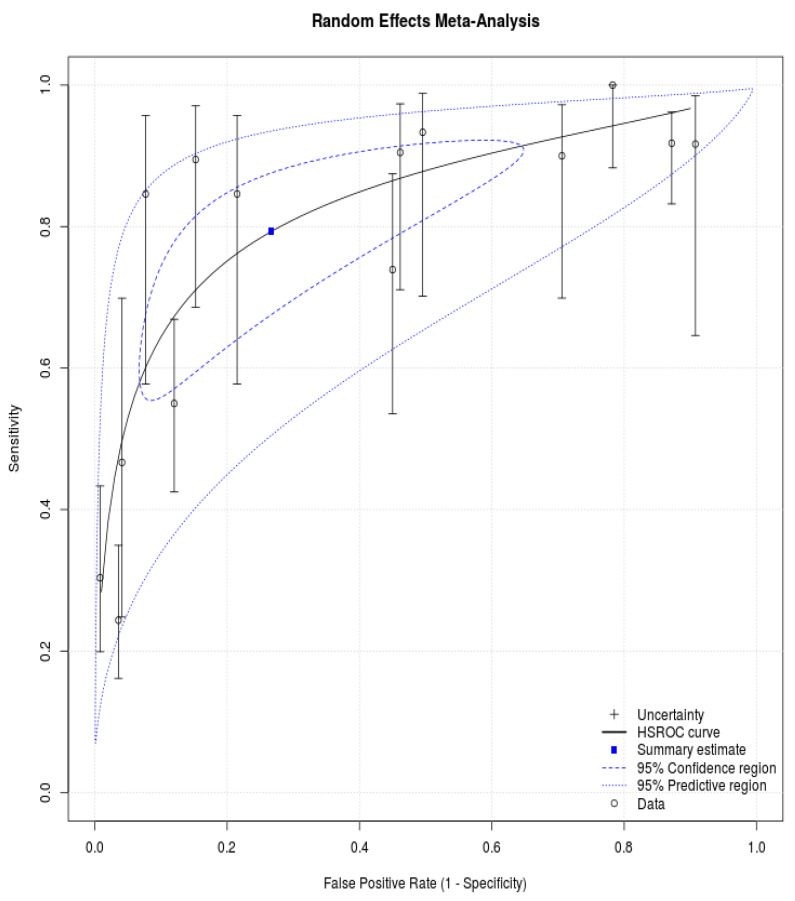
Summary receiver operating characteristic curves from all included studies. The dotted line represents the theoretical plot of a test with no discrimination. The black line represents the summary ROC curve that can be drawn through these values. The summary point estimate (blue spot) and its 95% confidence region around it (within the dotted lines). The height of the rectangles is proportional to the number of patients with traumatic bowel and mesenteric injuries across studies, the width of the rectangles corresponds to the number of patients without traumatic bowel and mesenteric injuries.

The bivariate model jointly synthesizes sensitivity and specificity to give summary estimates, which are drawn as the summary point on an SROC plot (Figure 3). Confidence and prediction regions plotted around the summary point enable joint inferences to be made about sensitivity and specificity. The summary point for the diagnostic accuracy can be estimated by the meta-analysis of these studies. This restriction reduces the data for meta-analysis from 14 studies. The summary sensitivity and specificity were 0.793 with 95% CI: 0.635–0.894, and 0.733 with 95% CI: 0.468–0.896, respectively. The diagnostic odds ratio is 10.53 with 95% CI: 5.56–19.96. Figure 3 shows this summary point with a 95% confidence region and a 95% prediction region. The confidence region is based on the CI around the summary point and indicates that, based on the available data, the prediction region around the summary point indicates the region where we would expect results from a new study in the future to lie, and is, therefore, wider than the confidence region as it goes beyond the uncertainty in the available data.

## 4. Discussion

CT is the first-line diagnostic modality for abdominal trauma, which can offer visceral organ information and determine further therapeutic options [1,34,35,36,37]. The shift to non-operative management of abdominal trauma has meant that how to select accurate patients to undergo conservative management is a crucial issue in current trauma care. The TBMI is still a surgical disease; however, increasing studies agree that non-operative strategy can apply to non-significant TBMI. This systematic review summarizes the studies’ results presenting the incidence and predicting ability of the free fluid in diagnosing surgically important TBMI.

The surgically important TBMI accounts for 10.7% who underwent abdominal CT with suspicions of TBMI. Free peritoneal fluid was one typical presentation in CT of TBMI and was considered highly associated with surgical TBMI [3,26,38,39,40,41]; however, several studies were still against this conclusion [33,42,43,44]. Lack of typical presentation, which leads to difficult diagnosis and the reader’s experience, became an important factor in determining the diagnosis accuracy. In our analysis, the summarized sensitivity of FF in diagnosing surgically important TBMI was 79.3%, with a high diagnostic odds ratio of about 10.53 (95% CI: 5.556–19.961). Rodriguez et al. had presented a systematic review of isolated free fluid on computed tomographic scans in blunt abdominal trauma and pointed out that in only 27% of patients did isolated free fluid need therapeutic laparotomy [38]. The review included articles earlier in the technology, and after decades, the development and evolution of the CT make it trustable to detect many intraperitoneal visceral injuries, including TBMI. We performed this review to emphasize the significance of this issue.

Several authors present the amount and location of FF as another important factor to determine the surgical importance [25,26,43]. Gonser-Hafertepen et al. presented that FF revealed more than one slice (5 mm) of one region in abdominal CT; the odds of therapeutic operation increased with significance [25]. Yu et al. also presented increasing isolated pelvic FF (41.5 mL vs. 2.3 mL) in males, indicating the positive laparotomy [43]. However, Mahmood et al. disagreed with this finding [26]. This finding might be related to surgical indication, patient selection, and hospital facility. The second issue arising in FF to diagnose TBMI was delayed perforation. Unlike other visceral trauma, postponed ischemia, necrosis, and perforation of the bowel cannot be predicted or diagnosed on the initial CT images [45]. The challenge persists with the risk of a delayed event that ischemia can lead to a delayed perforation, even with no FF in the initial CT [37,46]. The free fluid in TBMI is the most frequently missed lesion on the initial CT, especially in patients over 50 years old with ISS > 14 [47]. Close physical monitoring and repeated CT to increase the diagnostic performance was advised [42], and the following images can appropriately improve the diagnostic rate. Saku et al. noted the effectiveness of a CT scan at least eight hours after trauma in the detection of TBMI [48]. If patients suffer from new-onset abdominal pain and peritoneal signs, following the CT can differentiate the delayed TBMI. In a small group of trauma patients with questionable CT findings, a short-term (6–48 h) follow-up CT [5,42,49] may be accommodating in confirming or excluding bowel injury diagnosis. Although short-term imaging follow-up may be beneficial for some patients, it may delay discharge and expose patients to unnecessary radiation. Therefore, even though several authors claim the necessity and effectiveness of repeat CT, no particular comments or consensus was concluded for the CT programme plan’s protocols.

The study is the systematic review of surgical TBMI in contrast-enhanced CT. All available articles were revised and collected for evaluation of the contemporary practice. There were some limitations. First, we did not collect the non-English base literature, which might decrease another article’s possibility. However, our reviewers evaluated available abstracts; therefore, if the non-English article has an English abstract, we also enrolled in their data. Second, the definition of free fluid is variable, which might influence our data’s final distribution. Third, some manuscripts were published ten years earlier, and the technology and resolution of CT from then is disparate from the current status of these diagnostic tools. Therefore, selective bias cannot be avoided entirely. Fourth, no randomized controlled trial was done, and only a prospective observational study was done on this topic, which might affect the evidence strength not being decisive.

## 5. Conclusions

This study represents the most robust evidence (level 3a) to date that FF is not the absolute but an acceptable indicator for surgically important TBMI. However, there is still a need for randomized controlled trials to confirm.

## Figures and Tables

**Table 1:** The detail characteristics of included studies.

Author	Year	CT Technique	Equipment Type	TP	FN	FP	TN	*n*	Sensitivity	Specificity	Weigh Specificity	Weight Sensitivity
Molinelli et al. [20]	2018	120 kVp, 60–450 mAs, collimation: 1.2 mm × 40, pitch: 1.20.	Somatom Sensation 40 (Siemens)	18	2	24	10	54	0.9	0.294	6.494	7.625
Zingg et al. [24]	2018	120 kVp, 300 mA, table speed: 55 mm per rotation (0.8 s), pitch: 1.375	Light Speed VCT 64 Pro (GE Healthcare)	17	2	98	545	662	0.895	0.848	7.803	7.364
Faget et al. [14]	2015	120 kVp, 130 –700 mAs	LightSpeed VCT 16/ 64 detector scanner (GE Healthcare)	17	39	4	496	556	0.304	0.992	7.801	5.677
McNutt et al. [6]	2014	N/A	Somatom Sensation 40 (Siemens)	33	27	6	44	110	0.55	0.88	7.504	7.71
Gonser-Hafertepen et al. [25]	2014	N/A	64-slice he- lical MDCT (GE Healthcare)	11	2	11	132	156	0.846	0.923	7.7	6.606
Mahmood et al. [26]	2014	N/A	64-slice scanners (Siemens)	29	0	72	20	121	1	0.217	6.939	7.67
Park et al. [27]	2013	120 kVp, 180–380 mAs, nose index: 19, feed/rotation: 39.4 mm, pitch factor: 0.98	LightSpeed VCT (GE Healthcare) /Somatom Sensation 64 (Siemens)	19	59	1	27	106	0.244	0.964	7.135	6.672
Livingston et al. [28]	2001	N/A	N/A	7	8	83	1936	2034	0.467	0.959	7.834	5.847
Steenburg et al. [29]	2015	120 kVp, 300 mAs	64-slice helical MDCT scanner (Phillips Healthcare)	14	1	55	56	126	0.933	0.505	7.472	7.572
Yang et al. [30]	2016	N/A	64-detector helical CT scanner (Siemens)	67	6	68	10	151	0.918	0.128	6.985	7.829
Wu et al. [31]	2017	120 kVp, table speed: 11.25 mm/rotation, 5-mm prospective thickness, 5-mm pro- spective interval	LightSpeed QX/i Scanner (GE Healthcare)	11	2	20	73	106	0.846	0.785	7.573	7.297
Firetto et al. [17]	2018	N/A	Somatom Definition Flash, (Siemens)/LightSpeed (GE Healthcare)	19	2	6	7	34	0.905	0.538	5.618	7.693
Bhagvan et al. [32]	2013	N/A	High Speed Advantage (GE Healthcare)/ Volume Zoom (Siemens)/Sensation 16 (Siemens)	11	1	59	6	77	0.917	0.092	6.473	6.73
Be`ge et al. [33]	2014	120 kV, 200 mAs	Sensation 64 cardiac scanner (Siemens)	17	6	9	11	43	0.739	0.55	6.67	7.708

**Table 2:** The pooled performance of the current systematic review.

Parameter	Estimate	2.5% CI	97.5% CI
Sensitivity	0.793	0.635	0.894
Specificity	0.733	0.468	0.896
False Positive Rate	0.267	0.104	0.532
Diagnostic Odds Ratio	10.531	5.556	19.961
Log Likelihood Ratio + ve	2.972	1.47	6.006
Log Likelihood Ratio − ve	0.282	0.188	0.424

## Data Availability

The data is available by the request to the corresponding author.

## Supplementary Materials

The following are available online at https://www.mdpi.com/article/10.3390/diagnostics11112028/s1.

## References

[1] Liao C.-H., Hsieh F.-J., Chen C.-C., Cheng C.-T., Ooyang C.-H., Hsieh C.-H., Yang S.-J., Fu C.-Y. (2019). The Prognosis of Blunt Bowel and Mesenteric Injury—The Pitfall in the Contemporary Image Survey. J. Clin. Med..

[2] Khan I., Bew D., Elias D., Lewis D., Meacock L. (2014). Mechanisms of injury and CT findings in bowel and mesenteric trauma. Clin. Radiol..

[3] Bekker W., Kong V.Y., Laing G.L., Bruce J.L., Manchev V., Clarke D. (2018). The spectrum and outcome of blunt trauma related enteric hollow visceral injury. Ann. R. Coll. Surg. Engl..

[4] Malinoski D.J., Patel M.S., Yakar D.O., Green D., Qureshi F., Inaba K., Brown C.V.R., Salim A. (2010). A Diagnostic Delay of 5 Hours Increases the Risk of Death After Blunt Hollow Viscus Injury. J. Trauma Inj. Infect. Crit. Care.

[5] Fakhry S.M., Brownstein M., Watts D.D., Baker C.C., Oller D. (2000). Relatively Short Diagnostic Delays (<8 Hours) Produce Morbidity and Mortality in Blunt Small Bowel Injury: An Analysis of Time to Operative Intervention in 198 Patients from a Multicenter Experience. J. Trauma Acute Care.

[6] McNutt M.K., Chinapuvvula N.R., Beckmann N.M., Camp E.A., Pommerening M.J., Laney R.W., West O.C., Gill B.S., Kozar R.A., Cotton B.A. (2015). Early Surgical Intervention for Blunt Bowel Injury: The Bowel Injury Prediction Score (BIPS). J. Trauma Acute Care Surg..

[7] Chmátal P., Lacman J., Kupka P., Ryska M. (2007). The role of CT for indicating laparotomy in blunt abdominal trauma: Comparing CT results and surgical findings in a group of 101 patients. Prospective study. Perspect. Surg..

[8] Petrosoniak A., Engels P.T., Hamilton P., Tien H.C. (2013). Detection of significant bowel and mesenteric injuries in blunt abdominal trauma with 64-slice computed tomography. J. Trauma Acute Care Surg..

[9] Harmston C., Ward J.B.M., Patel A. (2018). Clinical outcomes and effect of delayed intervention in patients with hollow viscus injury due to blunt abdominal trauma: A systematic review. Eur. J. Trauma Emerg. Surg..

[10] Chereau N., Wagner M., Tresallet C., Lucidarme O., Raux M., Menegaux F. (2016). CT Scan and Diagnostic Peritoneal Lav-age: Towards a Better Diagnosis in the Area of Nonoperative Management of Blunt Abdominal Trauma. Injury.

[11] Drasin T.E., Anderson S., Asandra A., Rhea J.T., Soto J.A. (2008). MDCT Evaluation of Blunt Abdominal Trauma: Clinical Significance of Free Intraperitoneal Fluid in Males with Absence of Identifiable Injury. Am. J. Roentgenol..

[12] Yu J., Fulcher A.S., Turner M.A., Cockrell C., Halvorsen R.A. (2010). Blunt bowel and mesenteric injury: MDCT diagnosis. Abdom. Imaging.

[13] Romano S., Scaglione M., Tortora G., Martino A., Di Pietto F., Romano L., Grassi R. (2006). MDCT in blunt intestinal trauma. Eur. J. Radiol..

[14] Faget C., Taourel P., Charbit J., Ruyer A., Alili C., Molinari N., Millet I. (2015). Value of CT to Predict Surgically Im-portant Bowel And/or Mesenteric Injury in Blunt Trauma: Performance of a Preliminary Scoring System. Eur. Radiol..

[15] Hines J., Rosenblat J., Duncan D.R., Friedman B., Katz D.S. (2013). Perforation of the Mesenteric Small Bowel: Etiologies and CT Findings. Emerg. Radiol..

[16] Fakhry S.M., Allawi A., Ferguson P.L., Michetti C.P., Newcomb A.B., Liu C., Brownstein M.R. (2019). Blunt small bowel perforation (SBP): An Eastern Association for the Surgery of Trauma Multicenter Update 15 Years Later. J. Trauma Acute Care Surg..

[17] Firetto M.C., Sala F., Petrini M., Lemos A.A., Canini T., Magnone S., Fornoni G., Cortinovis I., Sironi S., Biondetti P.R. (2018). Blunt bowel and mesenteric trauma: Role of clinical signs along with CT findings in patients’ management. Emerg. Radiol..

[18] Virmani V., George U., MacDonald B., Sheikh A. (2013). Small-Bowel and Mesenteric Injuries in Blunt Trauma of the Ab-domen. Can. Assoc. Radiol. J..

[19] LeBedis C.A., Anderson S.W., Bates D.D.B., Khalil R., Matherly D., Wing H., Burke P.A., Soto J.A. (2016). CT imaging signs of surgically proven bowel trauma. Emerg. Radiol..

[20] Molinelli V., Iosca S., Duka E., De Marchi G., Lucchina N., Bracchi E., Carcano G., Novario R., Fugazzola C. (2018). Ability of Specific and Nonspecific Signs of Multidetector Computed Tomography (MDCT) in the Diagnosis of Blunt Surgi-cally Important Bowel and Mesenteric Injuries. Radiol. Med..

[21] Landry B.A., Patlas M.N., Faidi S., Coates A., Nicolaou S. (2016). Are We Missing Traumatic Bowel and Mesenteric Injuries?. Can. Assoc. Radiol. J..

[22] Moher D., Altman D.G., Liberati A., Tetzlaff J. (2011). PRISMA Statement. Epidemiology.

[23] Freeman S.C., Kerby C.R., Patel A., Cooper N.J., Quinn T., Sutton A.J. (2019). Development of an Interactive Web-Based Tool to Conduct and Interrogate Meta-Analysis of Diagnostic Test Accuracy Studies: MetaDTA. BMC Med. Res. Methodol..

[24] Zingg T., Agri F., Bourgeat M., Yersin B., Romain B., Schmidt S., Keller N., Demartines N. (2018). Avoiding Delayed Diagnosis of Significant Blunt Bowel and Mesenteric Injuries: Can a Scoring Tool Make the Difference? A 7-Year Retrospective Cohort Study. Injury.

[25] Gonser-Hafertepen L.N., Davis J.W., Bilello J.F., Ballow S.L., Sue L.P., Cagle K.M., Venugopal C., Hafertepen S.C., Kaups K.L. (2014). Isolated Free Fluid on Abdominal Computed Tomography in Blunt Trauma: Watch and Wait or Operate?. J. Am. Coll. Surg..

[26] Mahmood I., Tawfek Z., Abdelrahman Y., Siddiuqqi T., Abdelrahman H., El-Menyar A., Al-Hassani A., Tuma M., Peralta R., Zarour A. (2014). Significance of Computed Tomography Finding of Intra-Abdominal Free Fluid Without Solid Organ Injury after Blunt Abdominal Trauma: Time for Laparotomy on Demand. World J. Surg..

[27] Park M.-H., Shin B.S., Namgung H. (2013). Diagnostic performance of 64-MDCT for blunt small bowel perforation. Clin. Imaging.

[28] Livingston D.H., Lavery R.F., Passannante M.R., Skurnick J.H., Baker S., Fabian T.C., Fry D.E., Malangoni M.A. (2001). Free fluid on abdominal computed tomography without solid organ injury after blunt abdominal injury does not mandate celiotomy. Am. J. Surg..

[29] Steenburg S.D., Petersen M.J., Shen C., Lin H. (2014). Multi-detector CT of blunt mesenteric injuries: Usefulness of imaging findings for predicting surgically significant bowel injuries. Abdom. Imaging.

[30] Yang X.-Y., Wei M.-T., Jin C.-W., Wang M., Wang Z.-Q. (2016). Unenhanced Computed Tomography to Visualize Hollow Viscera And/or Mesenteric Injury After Blunt Abdominal Trauma: A Single-Institution Experience. Medicine.

[31] Wu C.-H., Wang L.-J., Wong Y.-C., Fang J.-F., Lin B.-C., Chen H.-W., Huang C.-C., Hung S.-C. (2011). Contrast-Enhanced Multiphasic Computed Tomography for Identifying Life-Threatening Mesenteric Hemorrhage and Transmural Bowel Injuries. J. Trauma Acute Care Surg..

[32] Bhagvan S., Turai M., Holden A., Ng A., Civil I. (2013). Predicting Hollow Viscus Injury in Blunt Abdominal Trauma with Computed Tomography. World J. Surg..

[33] Bege T., Chaumoître K., Leone M., Mancini J., Berdah S.V., Brunet C. (2013). Blunt bowel and mesenteric injuries detected on CT scan: Who is really eligible for surgery?. Eur. J. Trauma Emerg. Surg..

[34] Butela S.T., Federle M.P., Chang P.J., Thaete F.L., Peterson M.S., Dorvault C.J., Hari A.K., Soni S., Branstetter B.F., Paisley K.J. (2001). Performance of CT in Detection of Bowel Injury. Am. J. Roentgenol..

[35] Malhotra A.K., Fabian T.C., Katsis S.B., Gavant M.L., Croce M.A. (2000). Blunt Bowel and Mesenteric Injuries: The Role of Screening Computed Tomography. J. Trauma Inj. Infect. Crit. Care.

[36] Atri M., Hanson J.M., Grinblat L., Brofman N., Chughtai T., Tomlinson G. (2008). Surgically Important Bowel and/or Mesenteric Injury in Blunt Trauma: Accuracy of Multidetector CT for Evaluation. Radiology.

[37] Fang J.-F., Wong Y.-C., Lin B.-C., Hsu Y.-P., Chen M.-F. (2006). Usefulness of Multidetector Computed Tomography for the Initial Assessment of Blunt Abdominal Trauma Patients. World J. Surg..

[38] Rodriguez C., Barone J.E., Wilbanks T.O., Rha C.-K., Miller K. (2002). Isolated Free Fluid on Computed Tomographic Scan in Blunt Abdominal Trauma: A Systematic Review of Incidence and Management. J. Trauma Inj. Infect. Crit. Care.

[39] Ng A.K.T., Simons R.K., Torreggiani W.C., Ho S.G.F., Kirkpatrick A.W., Brown D.R.G. (2002). Intra-abdominal Free Fluid without Solid Organ Injury in Blunt Abdominal Trauma: An Indication for Laparotomy. J. Trauma Inj. Infect. Crit. Care.

[40] Cho H.S., Woo J.Y., Hong H.-S., Park M.H., Ha H.I., Yang I., Lee Y., Jung A.Y., Hwang J.-Y. (2013). Multidetector CT Findings of Bowel Transection in Blunt Abdominal Trauma. Korean J. Radiol..

[41] Hsu Y.-P., Liao C.H. (2016). Isolated Free Fluid without Pneumoperitoneum on Computed Tomography in Blunt Abdominal Trauma: Laparotomy Better Based on Imaging Finding and Clinical Presentation. J. Trauma Treat..

[42] Lannes F., Scemama U., Maignan A., Boyer L., Beyer-Berjot L., Berdah S.V., Chaumoître K., Leone M., Bège T. (2019). Value of Early Repeated Abdominal CT in Selective Non-Operative Management for Blunt Bowel and Mesenteric Injury. Eur. Radiol..

[43] Yu J., Fulcher A.S., Wang D.-B., Turner M.A., Ha J.D., McCulloch M., Kennedy R.M., Malhotra A.K., Halvorsen R.A. (2010). Frequency and Importance of Small Amount of Isolated Pelvic Free Fluid Detected with Multidetector CT in Male Patients with Blunt Trauma. Radiology.

[44] Ormsby E.L., Geng J., McGahan J.P., Richards J.R. (2005). Pelvic free fluid: Clinical importance for reproductive age women with blunt abdominal trauma. Ultrasound Obstet. Gynecol. Off. J. Int. Soc. Ultrasound Obstet. Gynecol..

[45] Kim H.C., Shin H.C., Park S.J., Park S.I., Kim H.H., Bae W.K., Kim I.Y., Jeong D.S. (2004). Traumatic Bowel Perforation: Analysis of CT Findings according to the Perforation Site and the Elapsed Time since Accident. Clin. Imaging.

[46] Raharimanantsoa M., Zingg T., Thiery A., Brigand C., Delhorme J.-B., Romain B. (2017). Proposal of a new preliminary scoring tool for early identification of significant blunt bowel and mesenteric injuries in patients at risk after road traffic crashes. Eur. J. Trauma Emerg. Surg..

[47] Lawson C.M., Daley B.J., Ormsby C.B., Enderson B. (2011). Missed Injuries in the Era of the Trauma Scan. J. Trauma Inj. Infect. Crit. Care.

[48] Saku M., Yoshimitsu K., Murakami J., Nakamura Y., Oguri S., Noguchi T., Ayukawa K., Honda H. (2006). Small Bowel Perforation Resulting from Blunt Abdominal Trauma: Interval Change of Radiological Characteristics. Radiat. Med..

[49] Sharma O.P., Oswanski M.F., Singer D., Kenney B. (2004). The Role of Computed Tomography in Diagnosis of Blunt Intestinal and Mesenteric Trauma (BIMT). J. Emerg. Med..

